# Trait impulsivity and impaired prefrontal impulse inhibition function in adolescents with internet gaming addiction revealed by a Go/No-Go fMRI study

**DOI:** 10.1186/1744-9081-10-20

**Published:** 2014-05-30

**Authors:** Wei-na Ding, Jin-hua Sun, Ya-wen Sun, Xue Chen, Yan Zhou, Zhi-guo Zhuang, Lei Li, Yong Zhang, Jian-rong Xu, Ya-song Du

**Affiliations:** 1 Department of Radiology, Ren Ji Hospital, School of Medicine, Shanghai Jiao Tong University, Shanghai 200127, PR China; 2 Department of Child & Adolescent Psychiatry Shanghai Mental Health Center, Shanghai Jiao Tong University, Shanghai 200030, PR China; 3 Ge Applied Science Laboratory, GE Healthcare, Shanghai, PR China

**Keywords:** Internet addiction, Response inhibition, fMRI, Go/No-Go

## Abstract

**Background:**

Recent studies suggest that Internet gaming addiction (IGA) is an impulse disorder, or is at least related to impulse control disorders. In the present study, we hypothesized that different facets of trait impulsivity may be specifically linked to the brain regions with impaired impulse inhibition function in IGA adolescents.

**Methods:**

Seventeen adolescents with IGA and seventeen healthy controls were scanned during performance of a response-inhibition Go/No-Go task using a 3.0 T MRI scanner. The Barratt Impulsiveness Scale (BIS)-11 was used to assess impulsivity.

**Results:**

There were no differences in the behavioral performance on the Go/No-Go task between the groups. However, the IGA group was significantly hyperactive during No-Go trials in the left superior medial frontal gyrus, right anterior cingulate cortex, right superior/middle frontal gyrus, left inferior parietal lobule, left precentral gyrus, and left precuneus and cuneus. Further, the bilateral middle temporal gyrus, bilateral inferior temporal gyrus, and right superior parietal lobule were significantly hypoactive during No-Go trials. Activation of the left superior medial frontal gyrus was positively associated with BIS-11 and Chen Internet Addiction Scale (CIAS) total score across IGA participants.

**Conclusions:**

Our data suggest that the prefrontal cortex may be involved in the circuit modulating impulsivity, while its impaired function may relate to high impulsivity in adolescents with IGA, which may contribute directly to the Internet addiction process.

## Introduction

Internet addiction (IA) is a newly identified condition that has attracted worldwide attention and involves loss of control over Internet use [1-4]. Internet gaming addiction (IGA), the most popular subtype of Internet addiction, has been extensively studied and is currently included in Section 3, the research appendix, of the Diagnostic and Statistical Manual version 5 (DSM-V) [5]. Converging evidence from diverse sources indicates that adolescents who compulsively use the Internet are at increased risk of suffering from a number of negative social, behavioral, and health consequences, including poor school performance, disorganized daily life, and poor personal relationships [6-11].

Impulsivity is viewed as a multifaceted trait that varies normally across the population, although high levels may predisposeto a range of dysfunctional behaviors, including addiction [12,13]. Individuals with addiction exhibit a cluster of symptoms involving impulsivity, which suggests a breakdown in the processes underlying inhibitory control. A core component of executive functioning is response inhibition, which is generally defined as the ability to adaptively suppress behavior when environmental contingences demand this [14]. Reduced response inhibition has been observed in several substance-dependent patient populations including alcohol- [15], cocaine- [16], and opioid- [17] dependent patients, and smokers [18,19]. Recent studies using self-report measures have also found impulsivity to be positively correlated with excessive computer game playing and excessive Internet use in general [20,21]. Some authors suggest that IA is an impulse disorder or is at least related to impulse control disorders [22-24]. Moreover, several neuroimaging studies have suggested diminished efficiency of response-inhibition processes in IA groups relative to healthy controls [25-27].

The main aims of the present study were to (1) investigate the IGA differences in response inhibition with behavioral and fMRI approaches using a Go/No-Go paradigm; (2) explore whether different facets of trait impulsivity are specifically linked to abnormal brain activation in IGA individuals; and (3) determine whether regions of abnormal brain activation are related to the Barratt Impulsiveness Scale-11 (BIS-11) and Chen Internet Addiction Scale (CIAS) scores, which represent the severity of impulsiveness and IGA, respectively. We hypothesized that IGA subjects would show reduced response inhibition compared with normal subjects. More specifically, on a behavioral level, IGA subjects would make more mistakes when they had to inhibit their response to infrequent No-Go stimuli, while on a functional level, some brain regions would be abnormally activated during No-Go trials in IGA subjects compared with controls.

## Materials and methods

### Subjects

All subjects were recruited from the Department of Child and Adolescent Psychiatry of Shanghai Mental Health Center. Seventeen subjects whose behaviors corresponded to DSM-IV criteria for IGA according to the modified diagnostic questionnaire for Internet addiction (i.e., the YDQ) criteria by Beard and Wolf [22] were imaged. Seventeen age- and gender-matched healthy individuals with no personal or family history of psychiatric disorders were also imaged as the control group. Both groups were required to have no history of head injuries or other major neurological disorders, be free of severe medical or surgical illnesses, be free of diagnoses such as schizophrenia, major depression with psychotic features, bipolar disorder, or substance use disorder according to an interview performed by an experienced psychiatrist, and not be treated with any medications or psychotherapy. All the IGA subjects and control groups were right-handed, and no subjects smoked.

The diagnostic questionnaire for Internet addiction was adapted from DSM-IV criteria for pathological gambling by Young [28]. Young’s Diagnostic Questionnaire (YDQ) consisting of eight "yes" or "no" questions was translated into Chinese. It includes the following questions: (1) Do you feel absorbed in the Internet (remember previous online activity or the desired next online session)? (2) Do you feel satisfied with Internet use if you increase your amount of online time? (3) Have you failed to control, reduce, or quit Internet use repeatedly? (4) Do you feel nervous, temperamental, depressed, or sensitive when trying to reduce or quit Internet use? (5) Do you stay online longer than originally intended? (6) Have you taken the risk of losing a significant relationship, job, educational, or career opportunity because of the Internet? (7) Have you lied to your family members, therapist, or others to hide the truth of your involvement with the Internet? (8) Do you use the Internet as a way of escaping from problems or of relieving an anxious mood (e.g., feelings of helplessness, guilt, anxiety, or depression)? Young asserted that five or more "yes" responses to the eight questions indicate a dependent user. Later, Beard and Wolf [23] modified the YDQ criteria. Respondents who answered "yes" to questions 1 through 5 and at least any one of the remaining three questions were classified as suffering from Internet addiction.

A basic information questionnaire was used to collect demographic information such as gender, age, final year of schooling completed, and hours of Internet use per week. A sub-group who were addicted to violent games (or similar) was then recruited as the IGA group. The study was approved by the Ethics Committee of Ren Ji Hospital, School of Medicine, Shanghai Jiao Tong University. Participants and their parents or legal guardians were informed of the aims of our study before MRI examinations, and written informed consent was obtained from the parents or guardians of each participant.

### Behavioral and personality assessments

Before scanning, four questionnaires were used to assess the participants’ behavioral and personality features: the Chen Internet Addiction Scale (CIAS) [29], the Self-Rating Anxiety Scale (SAS) [30], the Self-rating Depression Scale (SDS) [31], and the Barratt Impulsiveness Scale-11 (BIS-11) [32]. All questionnaires were initially constructed in English and then translated into Chinese.

### BIS-11

BIS-11 is a questionnaire on which participants rate their frequency of several common impulsive or non-impulsive behaviors/traits on a scale from 1 (rarely/never) to 4 (almost always/always). BIS-II consists of 30 items and can be divided into three subscales. Scoring yields a total score and the three subscale scores are derived by factor analysis: attention (rapid shifts and impatience with complexity), motor (impetuous action), and non-planning (lack of future orientation) [32]; higher scores signify higher impulsivity.

### Go/No-Go task

Participants completed a Go/No-Go Paradigm previously used for functional imaging of tic disorders (TD) [33], which consisted of seven blocks, with four task blocks and three fixation blocks in alternating order. Before the experimental trial began, instructions were displayed on the screen. The task required subjects to monitor a visual display while single uppercase letters are presented one at a time (250 ms duration, 1000 ms intertrial interval) on a black background. Participants were instructed to press the response button as quickly as possible at the occurrence of every letter except the letter X. Non-X’s occurred 87% of the time, requiring a button press, and X’s occurred for the remainder (13%), requiring the withholding response. At fixed moments during the task, participants were given the opportunity to take a short break. To maintain accuracy during the whole task, participants also completed a brief practice run of the task before scanning to familiarize them with the task instructions. The stimuli procedure and behavioral data were collected using the E-prime software system (Edition 2.0; Psychology Software Tools Inc., Sharpsburg, NC, USA). The accuracy of the Go and No-Go conditions and the reaction time of the Go condition were analyzed.

### Image acquisition and preprocessing

All MR imaging was performed on a 3.0 T General Electric MR scanner (SignaHDxt; GE Healthcare, WI, USA) with a standard GR quadrature head coil. Tape and padding were used to restrict possible head movement and dampen the noise of the scanner. The MR sequence for functional imaging was a gradient-echo echo-planar imaging (EPI) sequence [flip angle = 90°, repetition time (TR) = 2500 ms, echo time (TE) = 30 ms, field of view (FOV) = 230 × 230 mm, matrix 64 × 64, thickness =4 mm]. The scan was performed during the Go/No-Go task. With regard to our younger subjects, to control the overall scan time and their coordination, participants only completed one run of the task that lasted 245 s and consisted of 112 Go trials intermixed with 16 No-Go trials. Several anatomical images were collected following the functional images during the GNG task, including (1)a high-resolution3D T1-weighted spoiled grass gradient recalled (SPGR) sequence [TR = 9.4 ms, TE = 4.6 ms, flip angle = 15°, FOV = 256 × 256 mm, 155 slices, 1.0 × 1.0 × 1.0 mm voxel size], (2) axial T1-weighted fast field echo sequences [TR = 331 ms, TE = 4.6 ms, FOV = 256 × 256 mm, 34 slices, 0.5 × 0.5 × 4 mm voxel size], and (3) axial T2-weighted turbo spin-echo sequences [TR = 3013 ms, TE = 80 ms, FOV = 256 × 256 mm, 34slices, 0.5 × 0.5 × 4 mm voxel size].

Structural brain MRI scans (T1-and T2-weighted images) were inspected by two experienced neuroradiologists. No gross abnormalities were observed in either group. DICOM (Digital Imaging and Communications in Medicine) images from the scanner were converted to analyze format and then the subsequent image preprocessing and statistical analysis was performed using SPM8 package (Wellcome Department of Cognitive Neurology, London, UK). Data from each subject were corrected for slice timing and each image was realigned for motion correction. The realigned datasets were normalized to Montreal Neurological Institute (MNI) space to remove any minor (subvoxel) motion-related signal change. A 6-mm full-width-half-maximum Gaussian kernel was then used for data smoothing.

### First-level fMRI analysis

A general linear model (GLM) was applied to identify blood oxygen level dependence (BOLD) activation in relation to separate event types. There were two types of trials: Go and No-Go. The GLM design matrix included two task-related regressors. The six head-movement regressors derived from the realignment stage (head-movement parameters) were also included as covariates of no interest. GLM was independently applied to each voxel to identify voxels that were significantly activated for the event types of interest. A high-pass filter (cut-off 128 s) was used to improve the detection efficiency by filtering out the low-frequency noise caused by physiological effects.

### Second-level group fMRI analysis

Second-level analysis was performed for the group level, treating inter-subject variability as a random effect. First, we determined the voxels showing a main effect in Go and No-Go trials within each group. Second, we tested for voxels that showed significant differences in BOLD signal during Go/No-Go effects between IGA and HC groups. Multiple comparison correction was performed using the AlphaSim program in the Analysis of Functional Neuroimages software package, as determined by Monte Carlo simulations. Statistical maps of the two-sample t-test were created using a combined threshold of *p* < 0.01 and a minimum cluster size of 40 voxels, yielding a corrected threshold of *p* < 0.05. Finally, to further interpret our data, we performed post hocanalyses on the clusters showing between-group differences. The relationship between the level of activation in these clusters and the CIAS and BIS-11 score was examined in the IGA group using Pearson’s correlation tests, but also accounting for variance of trait anxiety and depression scores. In addition, the linear correlation of the CIAS was also conducted while the model controlled for the BIS scores.

### Statistical analysis

All statistical analyses were performed with the Statistical Package for the Social Sciences (SPSS) version 19.0 for Windows (SPSS Inc., Chicago, IL, USA). Group differences in demographic variables, BIS-11, and CIAS scores were analyzed using the independent t-test and chi-square test. The correlation between abnormal brain activations and behavioral and personality measures in subjects with IGA was assessed using Pearson’s correlation analysis. For the behavioral data, a repeated measures ANOVA was used to analyze the accuracy of the Go/No-Go Task, with group (IGA and control) as the between-participant factor, and Go/No-Go (Go and No-Go) as the within-participant factor. Statistical significances were defined at the 0.05 level, two-tailed.

## Results

### Demographic and behavioral results

There were no significant differences in the distributions of age, gender, or education years between the two groups. However, the IGA subjects spent more time on the Internet per week (*p* = 0.001) than controls, and had higher scores than controls for CIAS (*p* < 0.001) and BIS-II (*p* < 0.001) total scores (Table 1).

**Table 1:** Demographic and clinical characteristics of the IGA and the control subjects

	**IGA group (n = 17)**	**Control group (n = 17)**	**p value**
	**(Mean ± SD)**	**(Mean ± SD)**	
Age (years)	16.41 ± 3.20	16.29 ± 2.95	0.912
Gender (M/F)	14/3	14/3	1.000
Education (yeas)	8.82 ± 3.09	9.53 ± 3.43	0.533
Time for internet use per week (hours)	27.29 ± 14.59	10.59 ± 11.71	0.001
Chen Internet Addiction Scale (CIAS)	65.82 ± 3.25	42.88 ± 9.68	<0.001
Self-Rating Anxiety Scale (SAS)	45.12 ± 7.41	43.22 ± 6.31	0.35
Self-rating depression scale (SDS)	50.76 ± 7.93	48.09 ± 5.89	0.34
BarrattImpulsivenessScale-11 (BIS-11)	62.71 ± 4.37	53.24 ± 5.36	<0.001

Abbreviation. SD: standard deviation. Two-sample t test was used for group comparisons but chi-square was used for gender comparison.

Note: IGA = Internet gaming addiction.

### Behavioral results

In the Go/No-Go task, the accuracy and reaction time of the Go condition and the accuracy of the No-Go trials were used as the measure of inhibition (reaction times were not available as responses were not made on No-Go trials). The performance on No-Go trials was significantly worse than that on Go trials (*p* < 0.05) in the IGA group, but no other comparisons were significantly different (Table 2). Thus, the Go/No-Go performance was not used in the subsequent analysis.

**Table 2:** Go/No-Go task performance of the IGA and the control subjects

	**Mean (SD)**		**P value**
	**IGA group (n = 17)**	**Control group (n = 17)**	
% correct No-Go trials	60.3(31.9)	67.6.(26.2)	0.09
% correct Go trials	98.3(3.2)	98.5(4.3)	0.80
Mean RT Go, msec^a^	357.48(142.51)	364.57(107.97)	0.09

Note: IGA = Internet gaming addiction; RT = reaction time.

## fMRI results

**Figure 1:**
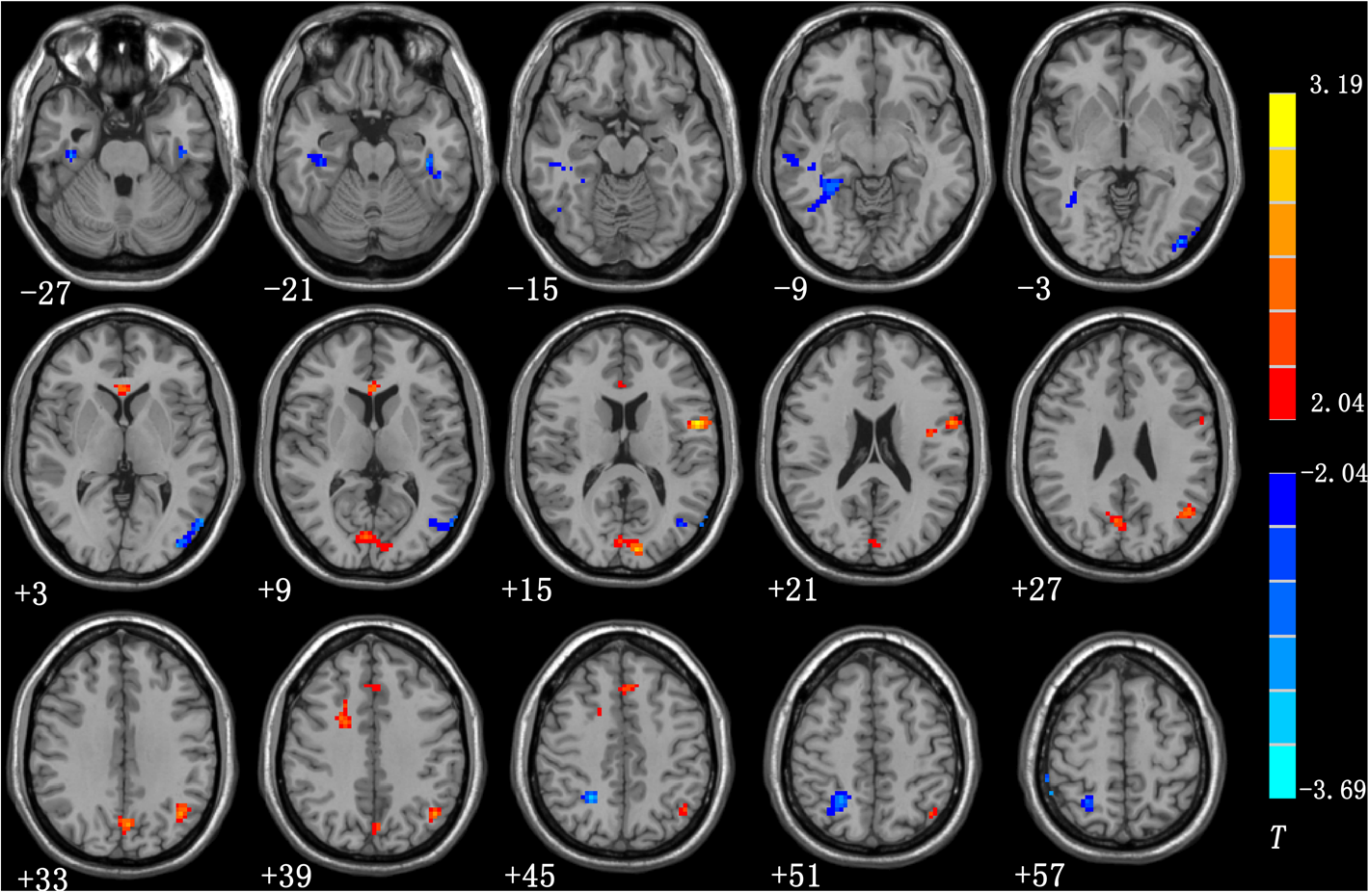
**Significant between-group differences of brain activation during No-Go trials between control subjects and IGA group.** Compared with the control group, the subjects with IGA exhibited hyperactivity during No-Go trails in the left superior medial frontal gyrus (BA8/6), right anterior cingulate cortex (ACC), right superior /middle frontal gyrus, left inferior parietal lobule , left precentral gyrus, as well as the left precuneus and left cuneus . However, several brain regions exhibited decreased activity during No-Go trails, including in bilateral middle temporal gyrus, bilateral inferior temporal gyrus, and right superior parietal lobule. (*p* < 0.05, AlphaSim-corrected). The t-score bars are shown on the right. Red indicates IGA > controls and blue indicates IGA < controls. Note: The left part of the figure represents the patients^,^ right side. IGA = Internet gaming addiction.

Our results indicate significant activation for response inhibition primarily in the bilateral mPFC, anterior cingulate cortex (ACC), and inferior frontal gyrus (IFG) in healthy controls (*p* < 0.01, FDR corrected). Moreover, when the analysis between the two groups was conducted, the IGA group was significantly hyperactive during the No-Go trials in the left superior medial frontal gyrus (BA8/6), right anterior cingulate cortex (BA 24), right superior /middle frontal gyrus, left inferior parietal lobule (BA39/40), left precentral gyrus, and left precuneus (BA7) and left cuneus (BA18) (Figure 1, Table 3); the bilateral middle temporal gyrus, bilateral inferior temporal gyrus, and right superior parietal lobule were significantly hypoactive during the No-Go trials.

**Table 3:** Brain regions showing significant difference of activation during No-Go trials in the IGA as compared to the controls

**Peak MNI coordinate region**	**Peak MNI coordinates **			**Number of cluster voxels**	**PeakTvalue**
	**X Y Z**				
1. left superior medial frontal gyrus (BA8/6)	0	26	43	42	2.415
2. rightanterior cingulated cortex (BA24)	3	26	7	45	3.060
3. right superior /middle frontal gyrus	21	11	43	42	2.634
4. left inferior parietal lobule	-42	-64	34	88	2.665
5. left precentralgyrus	-54	2	16	56	3.088
6. left cuneus (BA18)	-9	-91	16	85	2.828
7. left precuneus (BA7)	-3	-73	34	59	2.779
8. right middle temporal gyrus	39	-22	-26	63	-3.041
9. left inferior temporal gyrus	-42	-22	-23	56	-3.652
10. right inferior temporal gyrus	30	-43	-8	68	-2.776
11. left middle temporal gyrus	-60	-67	13	89	-3.320
12. right superior parietal lobule	24	-52	49	148	-3.336

(p < 0.05, AlphaSim-corrected).

Note: T > 0 indicated IGA > controls in abnormal brain activity during No-Go trials; T < 0 indicated IGA < controls in abnormal brain activity during No-Go trials.

Next, we calculated the linear correlations of the BIS and CIAS while accounting for variance of trait anxiety and depression scores. Activation of the left superior medial frontal gyrus was correlated with more severe total CIAS (r = 0.752, *p* = 0.001) and BIS-11(r = 0.63, *p* = 0.012) scores across IGA participants. In addition, the relationship between CIAS and activation in the left superior medial frontal gyrus survived (*p* < 0.01) while the model controlled for the BIS scores.

## Discussion

Impulse control impairments have been shown to be common to a number of addictive behaviors and may constitute a risk factor for drug abuse and dependence. Go/No-Go task-based fMRI have consistently shown activation of multiregion neural networks. It was recently reported that in the absence of performance deficits, current recreational drug users, whose predominant drug of choice was ecstasy, demonstrate hyperactive neural responses for successful inhibition in the right dorsolateral PFC (DLPFC), inferior frontal gyrus (IFG), and parietal lobule compared with well-matched controls [34]. Tapert et al. [35] reported no significant behavioral differences between a group of adolescent cannabis users and non-using controls when completing a Go/No-Go paradigm. However, the adolescent cannabis users appeared to recruit more frontal, parietal, and visual areas including the right dorsolateral prefrontal, bilateral medial frontal, bilateral inferior, and superior parietal lobules, and the right occipital gyrion No-Go trials. Consistent with previous studies, our results showed that in the absence of Go/No-Go performance deficits in the IGA group, hyperactive neural responses were found during No-Go trials in the anterior cingulate, dorsolateral prefrontal, premotor, and parietal cortices relative to well-matched healthy controls. However, our findings were mainly observed in the left hemisphere.

Recently, Hirose et al. [36] developed the efficiency index for evaluating the efficiency of response inhibition in the Go/No-Go tasks. In that study, the authors reported the presence of neural substrates of response inhibition in the left hemisphere that are modulated by efficiency, in addition to the well-established neural substrates of response inhibition in the right hemisphere. The correlation of left hemispheric dominance with efficiency suggests that the left hemisphere may play a supplementary role in response inhibition when the right hemisphere is already fully engaged. In particular, a recent study examining ICA of the stop signal task data showed that the right frontoparietal network implements attentional monitoring, whereas the left frontoparietal network implements response inhibition [37]. However, these possibilities are difficult to reconcile, and further studies are required to elucidate the left/right asymmetry of hemispheric contribution to response inhibition. Cognitive efficiency refers to the concept that performance may be optimized and may require less resources when a task is performed quickly [38]. Therefore, the IGA group’s hyperactivity in neural responses during No-Go trials in the absence of performance differences may indicate that greater demands were placed on the response-inhibition system in the IGA to maintain performance at levels comparable to the controls. Nevertheless, adolescents with IGA may have a diminished efficiency of response-inhibition processes.

With regards to Go/No-Go performance, no differences were observed between the IGA group and controls, which is consistent with one previous IA study [25] but not with others [39-41]. The cause of these discrepancies in response-inhibition tasks is unclear, and may reflect differences in sample-specific characteristics, task design, task difficulty, and participant intelligence. Indeed, for brain imaging studies, the absence of performance differences can be advantageous for removal of secondary performance-related effects (e.g., frustration) that may confound the group comparison [42]. Instead, the hyperactivity of the IGA in the absence of performance differences indicates that inhibition was more demanding and required greater levels of neuronal involvement. The ability of the users to use additional resources to maintain levels of performance comparable to controls suggests that their functional impairment may be subtler than we were able to determine.

The prefrontal cortex (PFC) is considered to play an important role in cognitive processes [43-45]. PFC impairments in addiction were also reported to result in executive dysfunction in the brain, which was considered to contribute directly to the addiction process [46]. Further, the dorsolateral prefrontal cortex was suggested to play a role in motivation and cognitive processes after receiving rewarding expectations [47,48], while the ventromedial prefrontal (VMPFC) region, including the orbitofrontal cortex (OFC), anterior cingulate cortex (ACC), and medial prefrontal cortex (MPFC), plays an important role in modulating impulsivity and aggression [49]. Moreover, the VMPFC has an anatomically intrinsic corticocortical network [50,51] and an extrinsic connection with the striatum, thalamus, brain stem, and limbic structures, including the amygdale, a circuit that is thought to contribute to impulsivity control [50,52]. Further, the inferior parietal cortex has extensive reciprocal connections with the prefrontal cortex [53]. These circuits appear to be critical for the executive control needed to guide goal-directed and stimulus-driven attention [54]. Some studies have hypothesized that the parietal cortex may play a role in regulating attention or withholding the motor response during response inhibition tasks [55,56]. In support, Hershey et al. [33] also suggested that parietal over-activity is either an underlying cause of poor inhibition or a response to failures of inhibition (e.g., increased attention or error monitoring following false alarms). The cognitive inefficiency observed in the IA group in the present study could arise from impaired "top-down" cognitive-control processes, which have been associated with increased neural activity in the ACC [57], and have been observed in nicotine-dependent participants performing the Stroop and working memory tasks [58-60]. Consistent with a previous study [27], we also found hyperactivity in the ACC. The precentral cortex is mainly involved in planning and executing movements [61,62]. Yuan et al. [63] reported increased cortical thickness in the left precentral cortex in late adolescence with online gaming addiction, and the cortical thicknesses of the left precentral cortex were correlated with duration of online gaming addiction. Taken together, their results suggested that the cortical thickness changes in the left precentral may be associated with the process of acquiring better playing skill going from a "rookie" to an "advanced player" [63].

We also found that the IGA group demonstrated hyperactive neural responses in the left medial frontal gyrus and left precuneus. As both the medial PFC and precuneus are part of the default-mode network (regions that are typically deactivated during active task performance [64], the increased deactivation in these regions in IGA group compared with controls suggests an impairment in turning off the default mode during attempts to inhibit. Impaired performance on attention demanding tasks has previously been associated with failure to deactivate the default-mode circuitry in both normal and clinical groups [65,66]. Our correlation analysis demonstrated that abnormal hyperactive function of the left superior medial frontal gyrus was positively associated with BIS and CIAS total score across IGA participants. These results suggest that the IGA subjects have diminished efficiency of response-inhibition processes, which may be explained, at least in part, by impaired functioning of the prefrontal cortex.

The IGA subjects were significantly hypoactive compared with the healthy controls in the bilateral middle temporal gyrus, bilateral inferior temporal gyrus, and right superior parietal lobule. These regions are suspected to be responsible for visual and auditory functions [67,68]. Online game playing requires players to stare at the computer screen and endure the sound of the game for long durations. Long-term hyperactivity of visual attention can impair the subject’s visual functions, while noise can impair their hearing abilities [69-71]. Further, Dong et al. [72] reported that IA subjects showed decreased regional homogeneity (ReHo) in the temporal, occipital, and parietal brain regions. Taken together, we suggest that long-time game playing (exposure to visual and auditory stimuli) may impair the player’s visual and auditory abilities.

There are several limitations that should be acknowledged in regard to this study. First, for our younger subjects, to control the overall scan time and their coordination, the Go/No-Go task was set to be short (4 min). Second, the absence of significant group differences in Go/No-Go behavior may indicate intact cognitive-control processes in the IGA group. However, fMRI can detect differences in cognitive processes that are too subtle to produce robust behavioral effects. Third, it is possible that the effects of Internet addiction may relate to the lack of time spent with family and friends and interacting in the "real world", as there is no clear evidence that supports the notion that changes in brain function are specific to the Internet activities rather than "lack of typical social interaction". Finally, the present study only revealed the current mental states of Internet addiction sufferers, while we were unable to determine the causal relationship between Internet addiction and the impaired executive control ability. Future studies are required to further assess executive control ability.

## Conclusion

Our data suggest that the prefrontal cortex may be involved in the circuit modulating impulsivity, and that its impaired function may underlie the high impulsivity in adolescents with IA, which may contribute directly to the Internet addiction process.

## Competing interests

The authors do not have an affiliation with or financial interest in any organization that might pose a conflict of interests.

## Authors’ contributions

Authors YZ, JS, JX and YD conceived and designed the experiments. Authors WD, JS, YS, XC, LL and ZZ performed the experiments. Authors WD, YZ, YS and XC analyzed the data. Authors WD, JS, YZ and YD wrote the paper. All authors read and approved the final manuscript.
